# Targeted Delivery of Neutralizing Anti-C5 Antibody to Renal Endothelium Prevents Complement-Dependent Tissue Damage

**DOI:** 10.3389/fimmu.2017.01093

**Published:** 2017-09-06

**Authors:** Paolo Durigutto, Daniele Sblattero, Stefania Biffi, Luca De Maso, Chiara Garrovo, Gabriele Baj, Federico Colombo, Fabio Fischetti, Antonio F. Di Naro, Francesco Tedesco, Paolo Macor

**Affiliations:** ^1^Department of Life Sciences, University of Trieste, Trieste, Italy; ^2^Institute for Maternal and Child Health-IRCCS “Burlo Garofolo”, Trieste, Italy; ^3^Dipartimento Universitario Clinico di Scienze Mediche, Chirurgiche e della Salute, University of Trieste, Trieste, Italy; ^4^ADIENNE Pharma & Biotech, Lugano, Switzerland; ^5^IRCCS Istituto Auxologico Italiano, Milan, Italy

**Keywords:** complement system, ischemia/reperfusion injury, targeted antibody-based therapy, *ex vivo* model, *in vivo* model

## Abstract

Complement activation is largely implicated in the pathogenesis of several clinical conditions and its therapeutic neutralization has proven effective in preventing tissue and organ damage. A problem that still needs to be solved in the therapeutic control of complement-mediated diseases is how to avoid side effects associated with chronic neutralization of the complement system, in particular, the increased risk of infections. We addressed this issue developing a strategy based on the preferential delivery of a C5 complement inhibitor to the organ involved in the pathologic process. To this end, we generated Ergidina, a neutralizing recombinant anti-C5 human antibody coupled with a cyclic-RGD peptide, with a distinctive homing property for ischemic endothelial cells and effective in controlling tissue damage in a rat model of renal ischemia/reperfusion injury (IRI). As a result of its preferential localization on renal endothelium, the molecule induced complete inhibition of complement activation at tissue level, and local protection from complement-mediated tissue damage without affecting circulating C5. The *ex vivo* binding of Ergidina to surgically removed kidney exposed to cold ischemia supports its therapeutic use to prevent posttransplant IRI leading to delay of graft function. Moreover, the finding that the *ex vivo* binding of Ergidina was not restricted to the kidney, but was also seen on ischemic heart, suggests that this RGD-targeted anti-C5 antibody may represent a useful tool to treat organs prior to transplantation. Based on this evidence, we propose preliminary data showing that Ergidina is a novel targeted drug to prevent complement activation on the endothelium of ischemic kidney.

## Introduction

The complement (C) system is an important humoral effector of innate immunity and is widely distributed in the circulation and at extravascular sites where it often provides the first line of defense against invading pathogens (1). C also plays a crucial role in maintaining homeostasis by contributing to clear apoptotic and necrotic cells, to remove immune complexes and to modulate adaptive immune responses (2). These functions are usually fulfilled by biologically active products released as a result of C activation that act promoting opsonization, inflammation, and direct cell cytotoxicity. The effector molecules or complexes, however, are not selective for the targets to neutralize, whether foreign or altered self, and may easily diffuse out into surrounding tissues and attack bystander cells. Under normal circumstances, undesired effects of the C system are prevented by soluble and cell-bound regulators that inhibit its activation at various steps of the C sequence (1). Unrestrictive C activation is the result of either over-activation due to excessive amount of triggering factors or the consequence of defective or dysregulation of C regulatory proteins. Several pathological conditions, including autoimmune diseases and more generally inflammatory disorders, are associated with C activation leading to the release of biologically active products, which can cause extensive tissue destruction.

Different therapeutic strategies have been developed to prevent C-mediated cell and tissue damage using neutralizing antibodies or peptides. The only approved therapeutic molecules are the plasma-derived C1 inhibitor, indicated for the treatment of hereditary angioedema (3), and the C5-blocking antibody eculizumab (Soliris), currently used to treat patients with paroxysmal nocturnal hemoglobinuria (PNH) (4) and atypical hemolytic uremic syndrome (aHUS) (5). Other soluble inhibitors under development are Mubodina, a neutralizing miniantibody against C5 (6), compstatin and its analogs, peptides preventing C3 activation through the alternative pathway (7), mirococept, CR1 CCP1–3 fused with a membrane-targeting amphiphilic peptide (8) and others (see reviews by Ricklin and Lambris) (9, 10).

A problem that has not yet been solved in the control of C-mediated diseases is how to reduce and possibly avoid the side effects, which may be associated with chronic neutralization of the C system (11), in particular, the increased risk of common and opportunistic infections. Moreover, the high cost of long-term treatment of patients with these drugs to prevent C activation represents a major limitation to their clinical use.

We sought to solve these problems developing an alternative therapeutic approach consisting in the preferential delivery of the C inhibitor to the organ involved in the pathologic process. The kidney was selected as a target organ to protect from C-mediated damage given the broad range of renal diseases caused by C dysregulation, including antibody-mediated glomerulopathies, thrombotic microangiopathies, progressive kidney diseases, and ischemia/reperfusion injury (IRI) (12). To this purpose, we generated a recombinant protein (Ergidina^®^) obtained by fusing a cyclic-RGD peptide to a neutralizing antibody to C5 (Mubodina^®^) and tested its protective effect in a rat model of renal IRI. C activated through any one of the three pathways by danger-associated molecular patterns, neo-antigens, and immune complexes (13) is actively involved in IRI, inducing C-mediated cell lysis and tubule-interstitial injury (14), and C3a and C5a-dependent inflammatory response (15).

We now present data showing that Ergidina has a distinctive homing property for renal endothelial cells and is effective in controlling tissue damage caused by renal IRI.

## Materials and Methods

### Production of Recombinant Proteins

Anti C5 scFv antibody (6) was cloned, using BssH2 and NheI restriction sites, into pMB-SV5 vector (16) containing human IgG1 Fc region to produce the scFv-Fc molecule called Mubodina (ADIENNE Pharma & Biotech). Ergidina was generated by replacing SV5 tag with the peptide RGD-4C (17). To this end, Mubodina was amplified with the following oligos pMBsense CTGCTTACTGGCTTATCG and pMB-RGD-anti GGTTTAAGCTTTTAGCCGCAGAAACAATCTCCTCGGCAGTCGCAGGCGCCTTTACCCGGGGACAGGGAGAG. The first oligo anneals on the vector pMB at the 5′ while the second anneals at the end of human CH3 region and introduce the RGD-4C sequence and the Hind III restriction site sequence. After PCR amplification, the fragments were cut with XbaI an Hind III restriction sites and cloned into pMB-SV5 vector. Finally, both Moubodina and Ergidina were subcloned into pUCOE (18) vector. All clones obtained were confirmed by sequencing.

Purified plasmid DNA was transfected with freestyle max reagent (Invitrogen) in CHO-S cells according to a standard protocol and the cells were grown in Pro-CHO 5 (Lonza). The recombinant scFv-Fcs were purified from cell-conditioned medium loaded on Protein A column and eluted with citric acid 0.1M pH 3. Fractions containing the recombinant proteins were selected by ELISA (6) and checked for purity by SDS-PAGE (19).

### Hemolytic Assay

The hemolytic activity of the classical pathway of the C system was evaluated incubating human serum with sensitized sheep red blood cells in the presence of different amount of purified recombinant antibodies, as previously described (6).

### Animals

Male Wistar rats weighing 240–270 g were obtained from a colony kept in the animal house at the University of Trieste. Male BALB/c mice weighing 20–24 g were purchased from Charles River Italy and maintained in our university facilities. The *in vivo* experiments were performed in compliance with the guidelines of the European (86/609/EEC) and Italian (D.L.116/92) laws, were approved by the Italian Ministry of Health and the Administration of the University Animal House, in line with NIH Guide for the care and use of laboratory animal, in order to minimize the number of animals used and their suffering.

### Evaluation of Ergidina^®^ Distribution using Time-Domain Near-Infrared Optical Imaging

Ergidina was labeled with N-hydroxysuccinimmide ester of the cyanine 5.5 (Cy5.5. Amersham Biosciences: Fluorolink Cy5.5 Monofunctional Dye 5-pack) following a previously reported procedure (19).

BALB/c mice received 0.05 mg of Ergidina labeled with 1 nmol of Cy5.5 in the tail vein. A small-animal time-domain Optix MX preclinical NIR-imager (Advanced Research Technologies) equipped with a pulsed laser diode and a time-correlated single photon counting detector was used in this study for the *in vivo* and *ex vivo* evaluation of labeled Ergidina distribution, as previously detailed (20).

### Binding of Ergidina to Ischemic Rat Kidney

Wistar rats were first anesthetized with sodium thiobarbital (Inactin, Sigma, 80 mg/kg) and then received i.v. 100–150 IU/kg of heparin (Ratiopharm, Germany).

The kidneys were excised and stored in ice for 24 or 48 h after perfusion with 5 ml of Celsior through a PE20 polyethylene catheter (Intramedic Clay-Adams, Sparks, MD, USA) inserted into the renal artery to remove blood.

Afterward, two groups of 18 kidneys each were infused with 1 ml of iso-osmotic physiologic sterile solution containing either 0.5 or 1 mg of Cy5.5-labeled Ergidina and stored at 4, 10, and 25°C for either 15 or 30 min. Three kidneys for each experimental condition were used and analyzed by time-domain optical imaging before and after washing with 20 ml of Celsior in order to quantify the amount of the initially injected and the remaining bound Ergidina after washing.

The kidneys were snap frozen in liquid nitrogen and kept at −80°C. Seven micrometer sections were also examined by confocal microscopy to confirm the data obtain using optical imaging.

### Confocal Microscopy

Seven micrometer sections were also analyzed by confocal microscopy to confirm data obtained using optical imaging. Sections were examined using a Nikon C1-SI confocal microscope (TE-2000U) equipped with a 20× and 60× oil immersion lens. Light was delivered to the sample with an 80/20 reflector. The system was operated with a pinhole size of one airy disk (30 nm). Electronic zoom was kept at minimum values for measurements to reduce potential bleaching, collecting series of optical images at 2 µm *z* resolution step size. The unstained tissue was visualized using the 488 nm light line of the Argon laser and a 515 dichroic mirror with a 30 nm band emission filter was used. The Cy5.5 was exited using the 640 nm light coming from a diode laser and the fluorescence collected using a 650 long pass mirror. All images were acquired in the linear intensity window and with no visible saturation points. Representative images are *z*-projection performed using standard deviation algorithm in ImageJ software (NIH).

### Model of Ischemia/Reperfusion in Rat

Two groups of six male Wistar rats were anesthetized with sodium thiobarbital (Inactin; 80 mg/kg) (Sigma) and underwent unilateral right nephrectomy following Pavone and Boonstra surgical procedure (21). After kidney excision, the animals were allowed to rest for 24 h, and then housed in metabolic cages for 24 h to collect urine 1 and 4 days after operation. Blood samples were drawn and analyzed to check glucose, blood cells, kidney, and liver functional parameters. Blood pressure was daily monitored using a tail sphygmomanometric device (22).

Two weeks after unilateral nephrectomy, all the rats underwent a second surgical intervention consisting in the exposition of left kidneys through a small flank incision and in the occlusion of both left renal artery and vein with a non-traumatic clamp for 45 min. At the end of the ischemic period, the clamp was released and the organ reperfused.

Before inducing ischemia, a PE20 polyethylen catheter (Intramedic Clay-Adams, Sparks, MD, USA) was inserted into the right femoral artery, gently pushed toward the iliac artery and abdominal aorta, and the tip of the catheter was finally placed at the origin of the left renal artery. Two groups of eight rats received 1 ml of Celsior containing 250 µg of either Ergidina or an unrelated miniantibody infused into the renal artery in a 5 min time.

Following IR procedure, the rats were housed in metabolic cages to collect daily excreted urine 1 and 4 days after operation, and blood samples were also obtained. The animals were sacrificed 4 days after surgery, and the kidneys were removed, embedded in OCT compound (Miles, Milan, Italy), snap frozen in liquid nitrogen, and kept at −80°C until used for immunofluorescence and histologic analysis.

Urinary proteins were analyzed using Bradfor solution (Sigma) and the serum creatinine level was quantified by Integrated System Dx 880 (Beckman Coulter).

### Immunofluorescence Analysis

Ergidina binding to kidney or heart samples was evaluated using frozen sections (7 µm) that had been incubated with the antibody (10 µg/ml) for 60 min at room temperature followed by FITC-labeled goat anti-human IgG (Aczon, Monte SanPietro, Bologna, Italy).

Tissue deposition of C3 was assessed by incubating frozen kidney 7 µm sections with 1:200 goat IgG anti-rat C3 (Cappel, ICN Biomedicals, Milan, Italy) for 60 min at room temperature followed by FITC-labeled rabbit anti-goat IgG at a 1:200 dilution (DAKO, Glostrup, Denmark) for additional 60 min at room temperature. A similar approach was used to evaluate the presence of C9, using rabbit IgG anti-rat C9 (a kind gift from Prof. P. Morgan, Cardiff, UK) at a 1:1,000 dilution, followed by FITC-labeled swine anti-rabbit IgG (DAKO) at a 1:40 dilution.

The fluorescence intensity was evaluated in 10 different randomly selected areas (0.07 mm^2^ each) of renal tissue. ImageJ image analysis software (National Institutes of Health) was used.

### Histomorphologic Evaluation

The excised kidneys were preserved in phosphate-buffered 10% formalin, embedded in paraffin wax, cut into thin sections (7 µm), and stained with hematoxylin and eosin.

Histopathological changes were analyzed for tubular necrosis, proteinaceous casts, and medullary congestion, as previously described (23). Tubular necrosis and extension of proteinaceous casts were both graded as follows: no damage (0), mild (+1, unicellular, patchy isolated damage), moderate (+2, damage less than 25%), severe (+3, damage between 25 and 50%), and very severe (+4, more than 50% damage). The degree of medullary congestion was defined as follows: no congestion (0), mild (+1, vascular congestion with identification of erythrocytes by +400 magnification), moderate (+2, vascular congestion with identification of erythrocytes by +200 magnification), severe (+3, vascular congestion with identification of erythrocytes by +100 magnification), and very severe (+4, vascular congestion with identification of erythrocytes by +40 magnification).

The tissue sections were analyzed in a blind fashion by two experienced observers.

### Statistical Analysis

The results were expressed as mean ± SD. Data were compared by ANOVA using *post hoc* analysis for paired multiple comparisons with Fisher’s corrected *t*-test. A non-parametric Mann–Whitney test was used to determine the significance of differences between tissue damage scores in the tested groups.

## Results

### Production and *In Vitro* Characterization of Ergidina

**Figure 1:**
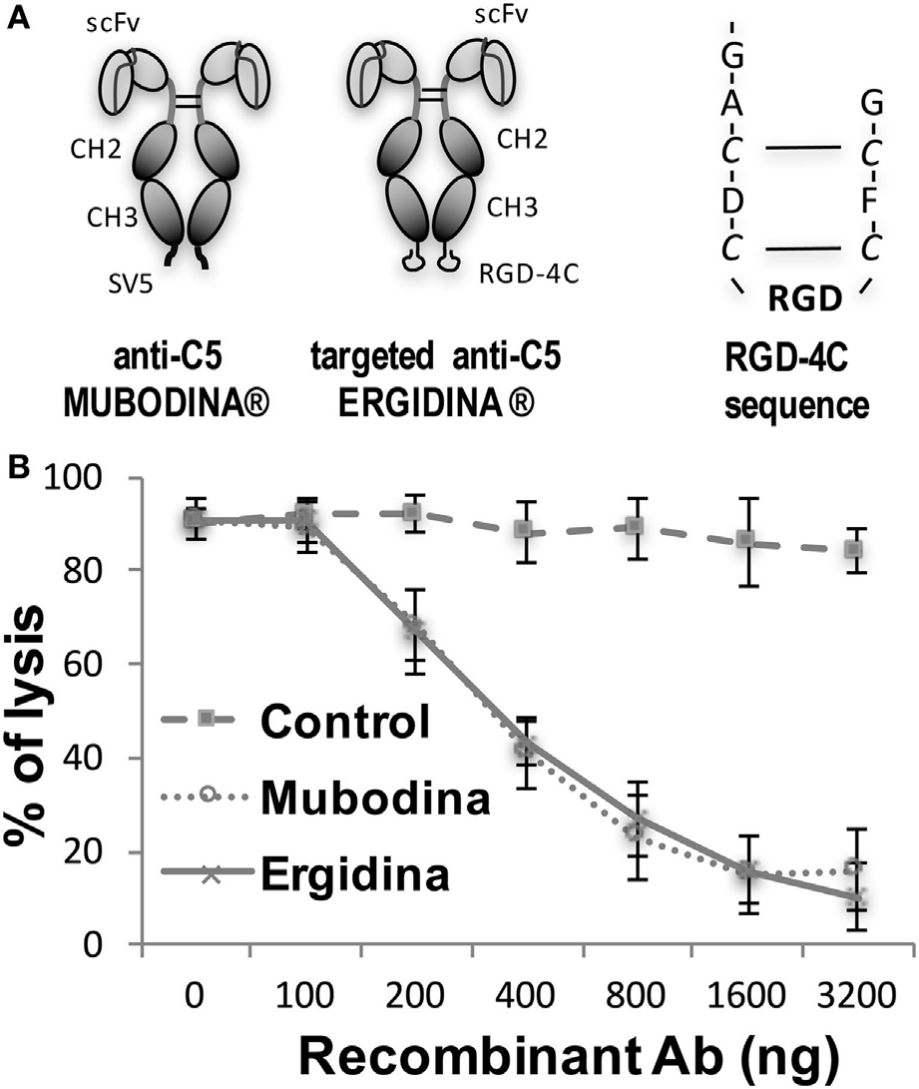
Structure and function of recombinant anti-C5 antibodies. **(A)** Schematic representation of Mubodina, Ergidina, and the peptide RGD-4C. **(B)** Inhibition of hemolytic activity by Mubodina and Ergidina. Increasing amounts of recombinant antibodies were mixed with normal human serum (1 µl) and incubated for 15 min at room temperature prior to addition to 50 µl of sensitized sheep erythrocytes. The serum hemolytic activity is presented as mean ± SD of percent values obtained in three different experiments.

We designed and cloned a novel recombinant antibody called Ergidina containing the neutralizing scFv to C5 (6, 24, 25) fused at the C terminal end to the Fc domains of human IgG1 (Hinge-CH2-CH3) to form Mubodina and the cyclic RGD-4C peptide (ACDCRGDCFCG) shown to bind avidly to the integrins αvβ3 and αvβ5 (26). A schematic picture of the two recombinant molecules, Mubodina and Ergidina, and the cyclic peptide RGD-4C used to generate Ergidina is presented in Figure 1A.

Analysis of Mubodina and Ergidina by SDS-PAGE and western blot revealed a major band of the expected size of 115–120 kDa, corresponding to the scFv-Fc dimers. Low amounts of monomers and degradation products were detected in all preparations of both Mubodina and Ergidina (Figure S1A in Supplementary Material). Moreover, Ergidina, examined by HPLC using SEC300 size exclusion column (Yarra), was eluted as a single peak corresponding to a protein of about 120 kDa (Figure S1B in Supplementary Material). Immunoenzymatic analysis showed that both miniantibodies were able to bind human C5, but failed to interact with human C3 (Figure S1C in Supplementary Material). We also tested the ability of Ergidina to neutralize C5 and to prevent C activation using a standard hemolytic assay. As shown in Figure 1B, Ergidina was found to be as efficient as the parent molecule Mubodina in inhibiting serum C hemolytic activity at similar concentration.

### *In Vivo* Distribution of Ergidina

**Figure 2:**
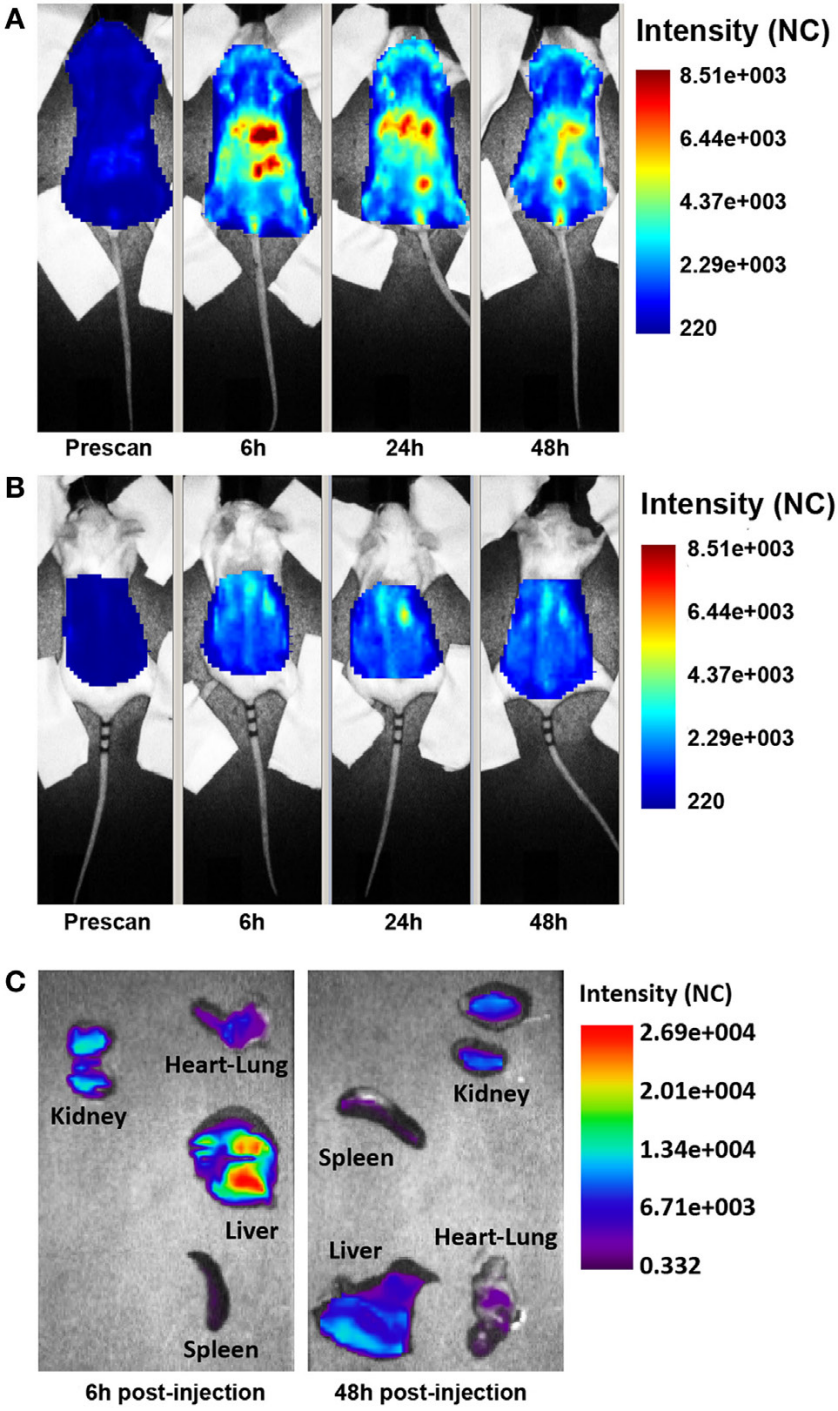
Biodistribution of Cy5.5-labeled Ergidina. BALB/c mice received i.v. 50 µg of Cy5.5-labeled Ergidina and were analyzed by optical imaging. **(A)** Whole-body scans of a representative mouse in supine position; fluorescence intensity images were acquired at the indicated time post-injection and are displayed as normalized counts (NC). **(B)** The animal was placed in prone position, fluorescence emission in regions of interest encompassing the kidneys was acquired at indicated times postinjection and normalized. **(C)** *Ex vivo* imaging of organs collected 6 or 48 h after Ergidina-Cy5.5 administration.

The distribution of the targeted anti-C5 recombinant antibody was first examined in healthy mice. The antibody (0.05 mg) labeled with 1 nmol Cy5.5 was injected intravenously (i.v.) into the tail vein, and its localization was examined by time domain optical imaging. Ergidina was diffusely distributed throughout the mouse body soon after i.v. administration followed, 6 h later, by a preferential accumulation in the kidney peaking at 24 h (Figure 2A). As already observed with the distribution pattern of other Cy5.5-labeled antibodies (19, 27), a proportion of the molecule was removed by the liver and the free dye was excreted through the kidney, thus explaining the visualization of fluorescent signals in the liver and bladder (Figures 2A,B). These data were confirmed by *ex vivo* analysis of the different organs performed 6 and 48 h after injection of the labeled molecule (Figure 2C). The pharmacokinetic profile of Ergidina was supported by confocal microscopy analysis of sections of different organs removed 6 h after antibody challenging that revealed specific near infrared fluorescent staining in the kidney and liver. The green fluorescence observed in both these organs was due to autofluorescence observed also in the lung that was examined as a control organ (Figure S2 in Supplementary Material).

### *Ex Vivo* Binding of Ergidina to Ischemic Kidney

**Figure 3:**
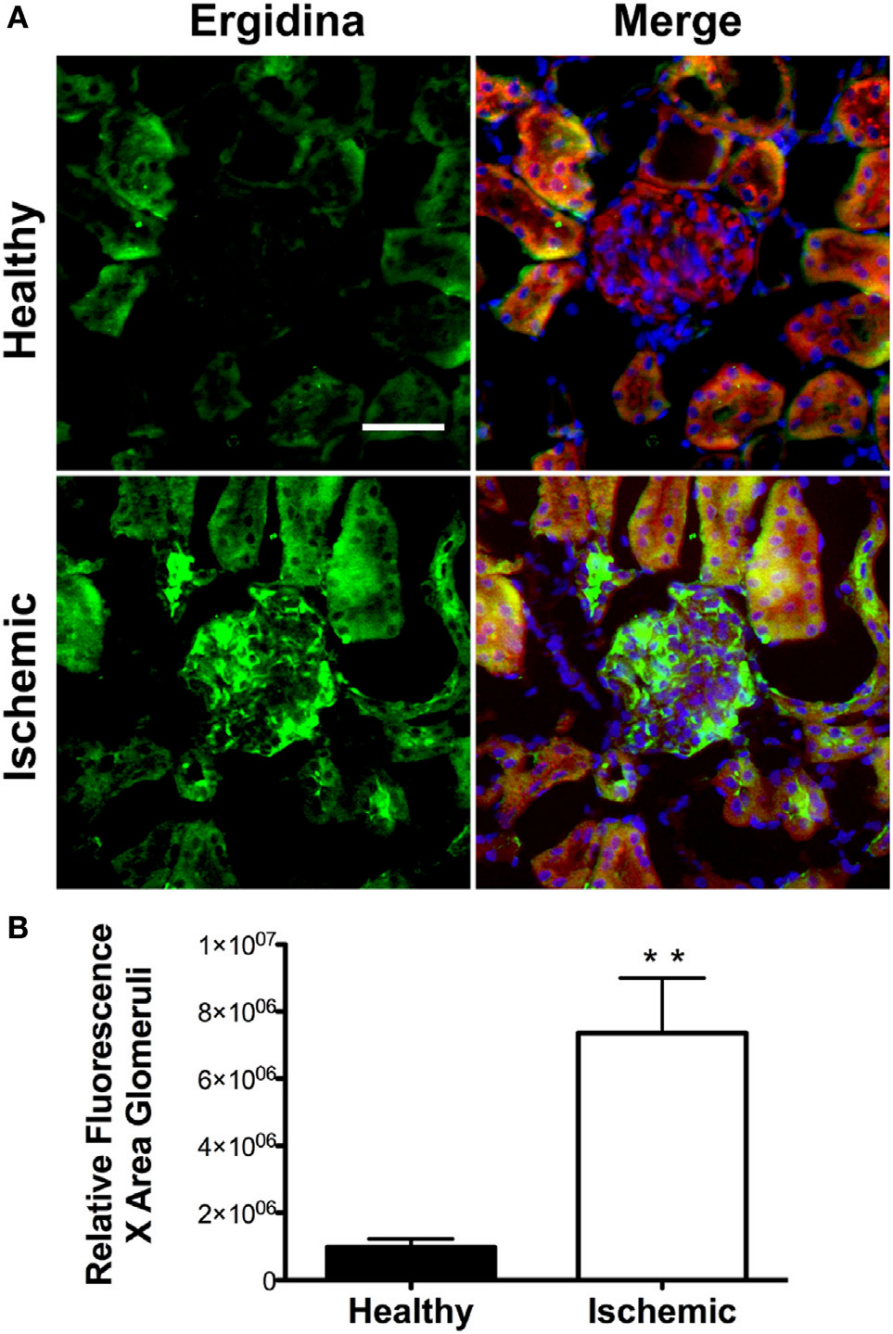
Immunofluorescence analysis of Ergidina deposition on ischemic kidney. **(A)** Representative images from section obtained from healthy or ischemic kidneys incubated with Ergidina, followed by FITC-labeled anti-human IgG. Nuclei were counterstained with DAPI and tissue was visualized by orange autofluorescence. Original magnification: 200×. Scale bar: 100 µm. **(B)** ImageJ software was used to determine the fluorescence intensity/glomerular area of the images for each experimental group. Values are the mean ± SD. **P < 0.001.

To investigate the extent of Ergidina deposition in isolated perfused rat kidney under various experimental conditions, the organ was removed from healthy animals and either fixed immediately or kept in ice for 24 h to mimic ischemic conditions prior to fixation in formalin. Tissue sections were then analyzed for antibody deposits. As shown in Figure 3A, Ergidina bound weakly to the endothelium and more strongly to the tubules of normal kidneys. Conversely, glomerular and vessel endothelium of ischemic kidney were heavily decorated by RGD-targeted antibody under ischemic conditions with levels of fluorescence intensity of glomeruli approximately sevenfold higher than that observed in control kidney (Figure 3B).

**Figure 4:**
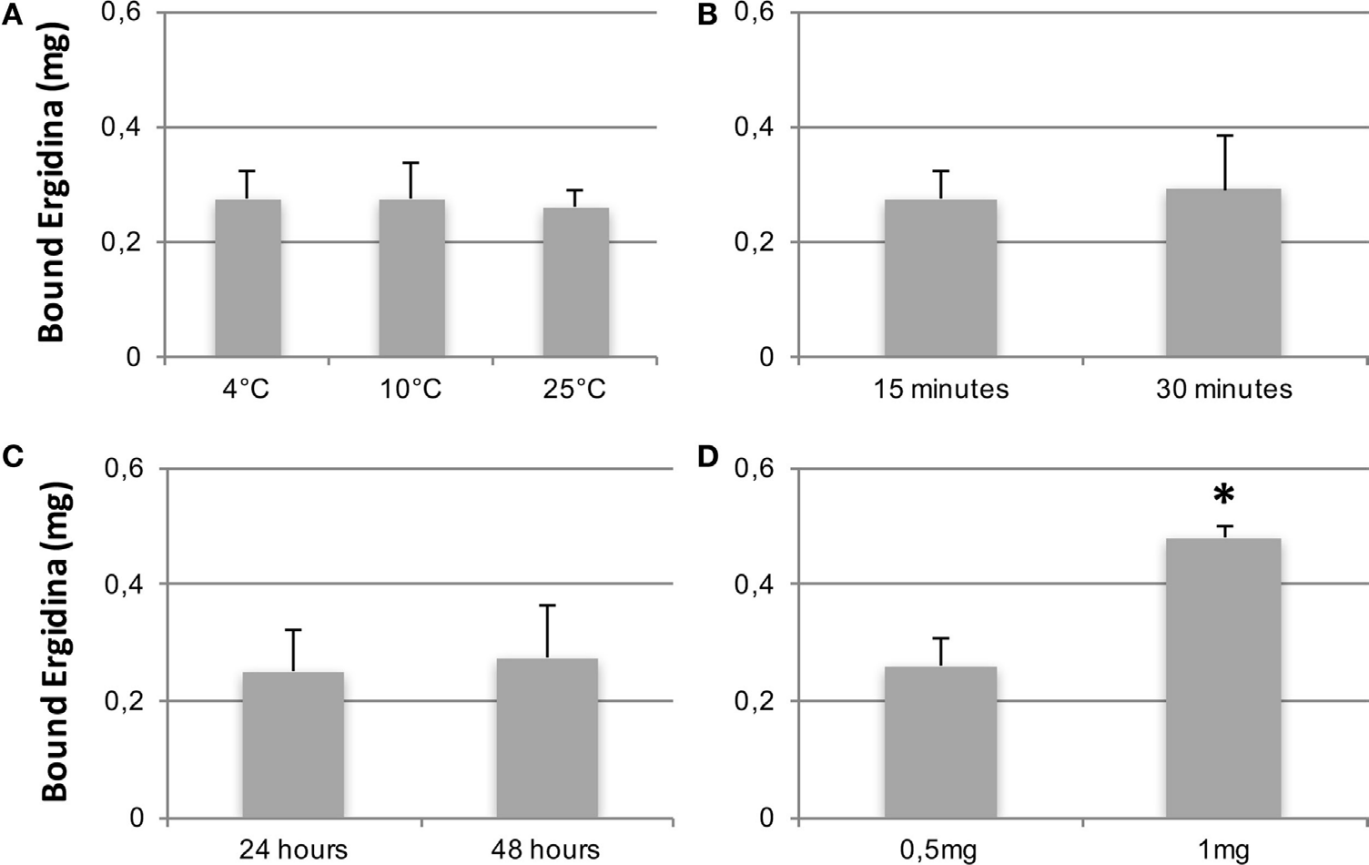
CCharacterization of Ergidina binding to ischemic kidneys. Rat kidneys were excised, incannulated, and perfused as described in Section “Materials and Methods.” Cy5.5-labeled Ergidina was injected through the renal artery. Each organ was visualized, washed, and analyzed again by time-domain optical imaging. Experimental conditions were maintained in all the groups except for the temperature of the binding **(A)**, the time of the binding **(B)**, the period of kidney ischemia **(C)**, or the amount of injected Ergidina **(D)**. Data are expressed as mean of the amount of bound antibody ± SD obtained from three organs per group. *P < 0.01.

We then tested the capacity of Ergidina to decorate renal endothelium of kidneys kept under different ischemic conditions. To this end, 0.5 mg of Cy5.5-labeled Ergidina was injected into the renal artery of surgically removed kidneys that had been washed and stored at 4°C for 24 h in Celsius^®^. The fluorescence intensity was assessed before and after organ perfusion using time-domain optical imaging. As shown in supplemental Figure S3, approximately half of the antibody was still bound to the renal vessels after incubation for 15 min at 4°C followed by perfusion to wash out unbound antibodies. This percentage remained essentially unchanged when the incubation temperature was raised to 10 and 25°C (Figure 4A). The binding of RGD-targeted antibody to the vascular endothelium of kidney kept at 4°C for 24 h occurred in a short period of time reaching a plateau at 15 min (Figure 4B). The percentage of bound antibody was slightly increased when the cold ischemia time was prolonged from 24 to 48 h. However, the difference was not statistically significant (Figure 4C).

**Figure 5:**
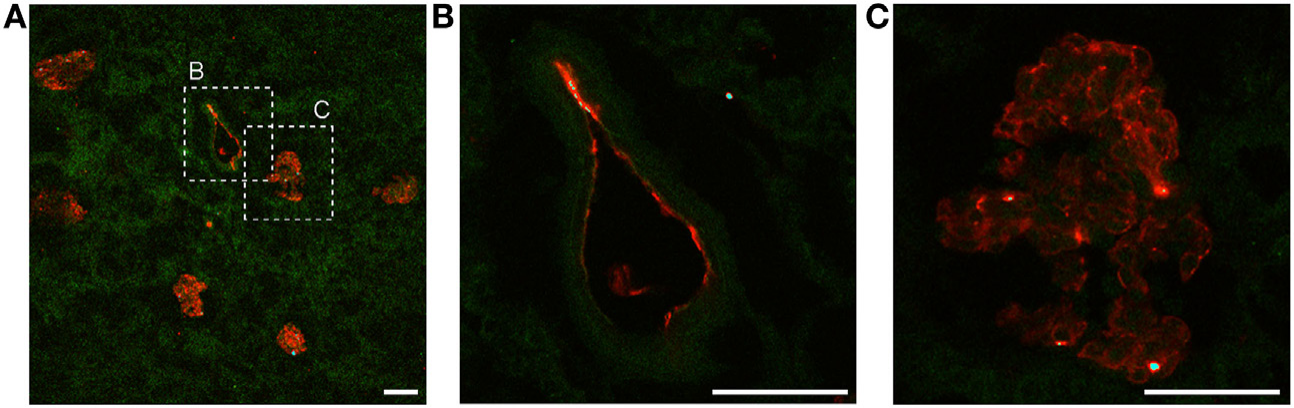
Ergidina localization in ischemic rat kidney. **(A)** Representative images from section obtained from ischemic kidneys perfused with cy5.5-labeled Ergidina. Tissue was evidenced by green autofluorescence. Magnification 200×. Close up images at high magnification (600×) of a **(B)** vessel and **(C)** glomerular structure showing specific staining. Scale bar: 50 µm.

The amount of Ergidina bound to the kidney stored at 4°C for 24 h was related to the dose of administered antibody and increased from 0.26 to 0.48 mg using doses of 0.5 and 1 mg, respectively (Figure 4D). The analysis of frozen sections of these organs treated with 0.5 mg of cy5.5-labeled Ergidina by confocal microscopy showed that the antibody was widely distributed on all glomeruli and extraglomerular vessels and was undetectable on renal tubules (Figure 5).

To assess whether the binding of Ergidina to surgically removed organs was restricted to the kidney, we analyzed isolated perfused rat heart stored at 4°C for 24 h. Frozen sections of this organ were incubated with Cy5.5-labeled Ergidina and examined by confocal microscopy. Figure S4 in Supplementary Material shows that the endothelium of ischemic heart is covered by RGD-guided anti-C5 antibody.

### Ergidina Prevents Tissue Damage in a Rat Model of Kidney Ischemia/Reperfusion

The finding that Ergidina preferentially accumulates in rat renal vessels *in vivo* suggested a potential use of the targeted antibody to prevent renal C-mediated damage. This hypothesis was tested in a rat model of kidney IRI described in Section “Materials and Methods.”

**Figure 6:**
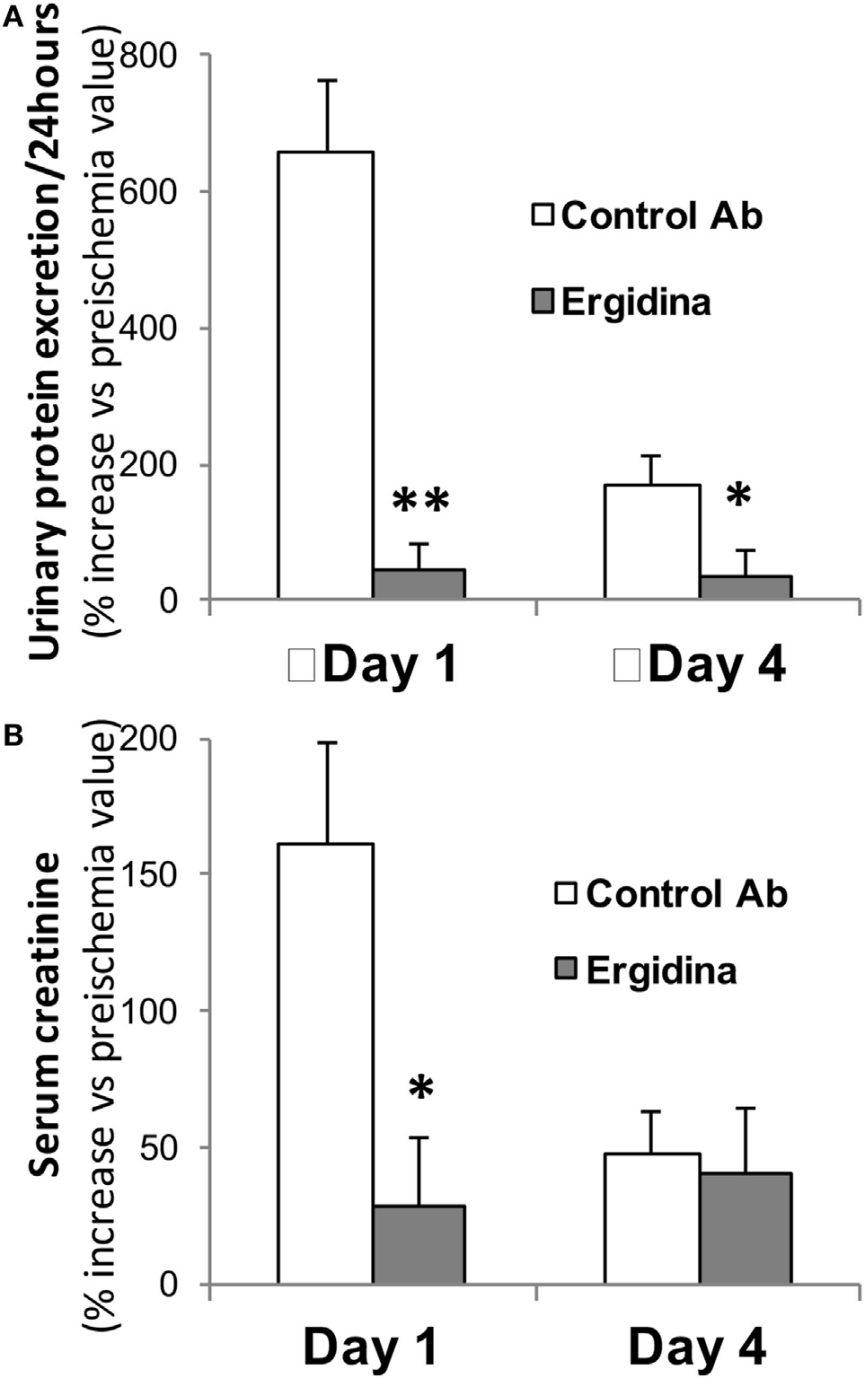
Effect of Ergidina in an ischemia/reperfusion injury model in rat kidney. Two groups of four rats received Ergidina or control Ab before inducing renal ischemia, as described in Section “Materials and Methods.” One or four days later, the animals were examined for **(A)** urinary protein excretion and **(B)** serum creatinine. Data are expressed as mean of the % increase vs preischemia value ± SD. *P < 0.01; **P < 0.001.

Prior to the ischemic injury, the animals received 0.25 mg of Ergidina found in the *ex vivo* experiments to fully cover the renal endothelium. After 45 min of ischemia, the kidney was reperfused and samples of serum and urine were collected 24 and 96 h later for analysis. The data reported in Figure 6 show clear signs of renal impairment in animals receiving the control antibody with marked increase in both protein excretion and serum creatinine level peaking 24 h after reperfusion. In contrast, Ergidina was able to completely prevent C-mediated damages, as revealed by the lack of significant changes in protein and creatinine concentrations that were comparable to those collected before IRI. The effect of Ergidina was still apparent 4 days after induction of ischemia despite the marked reduction in the protein and creatinine levels observed in the control group of rats.

**Figure 7:**
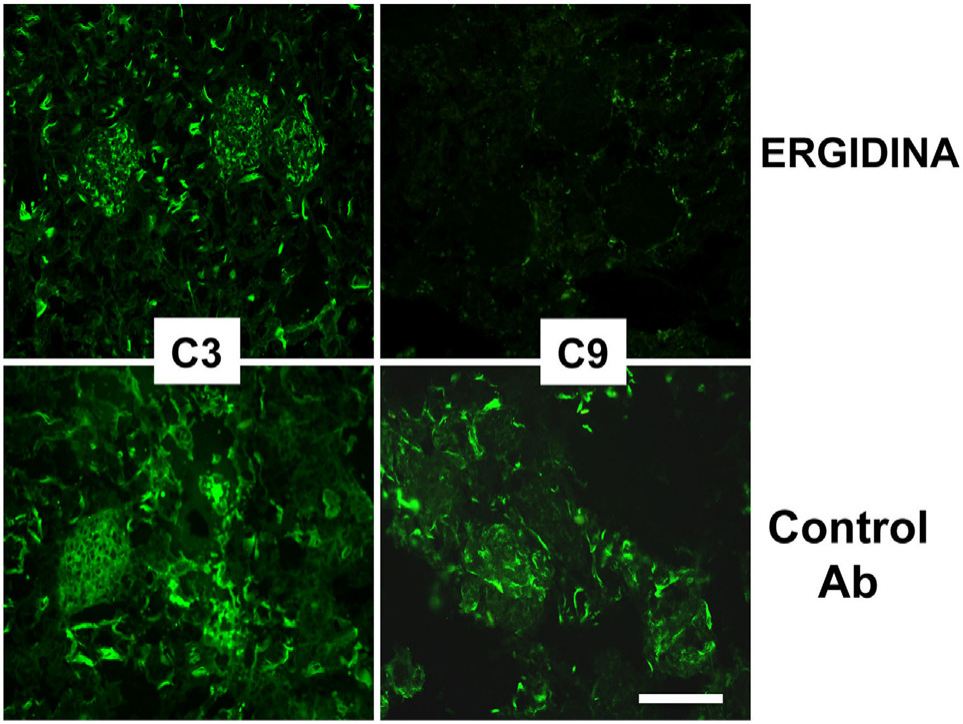
Immunofluorescence analysis of C deposition in kidney ischemia/reperfusion injury model. Deposition of C3 and C9 was analyzed in cryosections of organs obtained from rat treated with Ergidina or control antibody using specific goat polyclonal antibodies and FITC-labeled secondary antibody. Scale bar: 50 µm.

We also investigated the ability of Ergidina to control C-mediated tissue damage in ischemic kidneys removed from rats treated with the C5 neutralizing and control antibodies. A strong C activation was observed in the ischemic rat model that proceeds to completion of the reaction sequence as documented by C3 and C9 deposition in the kidneys of rats receiving an irrelevant antibody. As expected, administration of Ergidina to rats undergoing renal IRI did not prevent C3 deposition but resulted in the complete inhibition of C5 activation and an undetectable localization of C9 on glomeruli and vascular endothelium (Figure 7).

**Figure 8:**
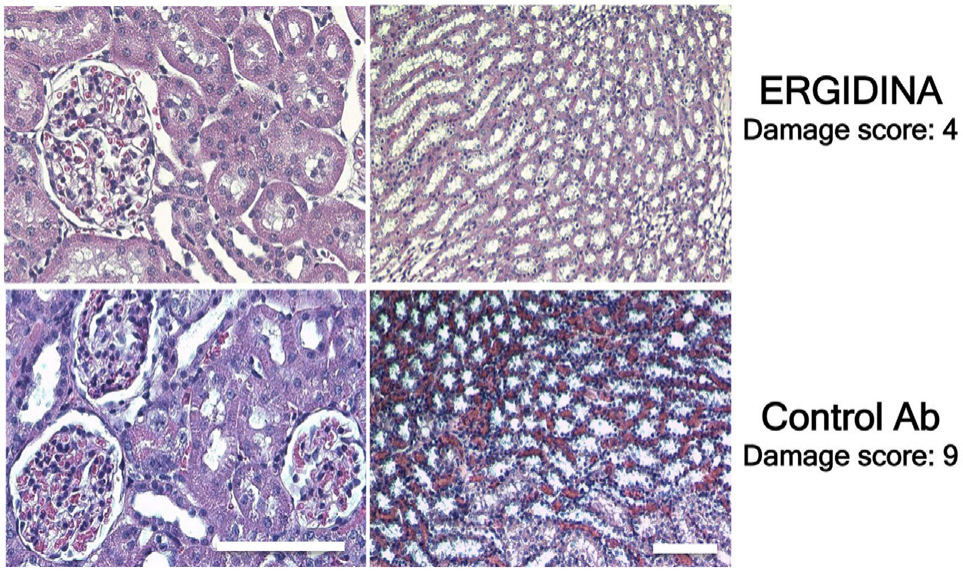
Histological analysis of treated ischemic kidneys. Photomicrographs of kidney sections obtained from a representative rat treated with Ergidina or control Ab and sacrified 4 days after ischemia/reperfusion injury. Note the extensive necrosis of tubular cells, some glomerular hypercellularity and mesangial hyperplasia in control Ab samples that were absent in Ergidina treated animals. Scale bar: 50 µm. A tissue damage score was determined as described in Section “Materials and Methods.” Values are the mean of four rats per group.

Histologic analysis of ischemic kidney of rats treated with the control antibody revealed extensive necrosis of tubular cells, and some degree of glomerular hypercellularity and mesangial hyperplasia (Figure 8). Injection of Ergidina proved to be effective in preventing tissue injury both at glomerular and tubular level, reducing the damage score to more than half compared to that observed in the control group of rats.

The dose of Ergidina used in this study was sufficient to fully inhibit C5 activation at renal level preventing tissue injury and increase in serum creatinine and urine protein levels, and was not associated with any overt sign of toxicity.

## Discussion

Efforts are being made to develop drugs that preferentially localize in organs and tissues undergoing a pathologic process mediated by effector molecules present in the circulation and in the body fluids. C has been largely implicated in several pathologic conditions and C neutralization has been shown to exert beneficial effect in preventing tissue and organs damage in both experimental and clinical settings (1, 2). For example, targeting C5 with the neutralizing antibody Eculizumab in humans has proved effective in controlling disease severity and progression in patients with PNH (4), aHUS (5), membranoproliferative glomerulonephritis (28), and antiphospholipid syndrome (29). Nevertheless, this therapeutic approach, though successful, is fraught with limitations due to the high cost and the possible side effects associated with generalized inactivation of the C system (30, 31). Our data indicate that this therapeutic strategy can be improved by developing a recombinant antibody that accumulates predominantly in the pathologic tissue.

To target endothelial cells with a neutralizing anti-C5 antibody, we used a recombinant human anti-C5 miniantibody recognizing C5 from human and other animal species (6) fused to the RGD-4C peptide at the Fc terminus. RGD-4C was selected for the study because this cyclic peptide offers several advantages over the linear peptide including increased structural stability, reduced susceptibility to degradation, and higher affinity for the target molecules (32).

Cyclic RGD has extensively been used in tumor-bearing mice to deliver imaging probes or drugs to tumor tissue for diagnostic and therapeutic purposes exploiting the ability of the peptide to recognize with high affinity αvβ3 integrin highly expressed in tumor vessels (33). The *in vivo* distribution of Ergidina observed in rats after i.v. administration suggests that RGD-4C can be used as a vehicle to deliver the neutralizing anti-C5 miniantibody to the kidney. The renal localization of Ergidina cannot be attributed to the clearance of the molecule, because its molecular weight of approximately 120 kDa is well above the size limit of the molecules filtering through the glomeruli under physiologic conditions. The weak fluorescent signal observed in the bladder is due to small amount of free fluorescent dye excreted in the urine (20, 27). A plausible explanation for the preferential homing of the neutralizing anti-C5 antibody to the kidney is that the recombinant molecule interacts with integrins expressing RGD-binding sites. Although αvβ3 and αvβ5 that bind RGD-4C peptide with high affinity are not expressed on quiescent endothelial cells, the mild staining of the vascular endothelium in normal kidney can possibly be justified by the presence of other integrins that bind less avidly RGD-4C, including α5β1 expressed on the endothelial cells of normal glomeruli (34, 35). The stronger immunofluorescence signal observed in the glomeruli of kidneys obtained from rats following IRI compared to untreated controls is consistent with the known ability of activated endothelial cells to express αvβ3 (35). This is also supported by the finding that RGD binding sites colocalize with αvβ3 on the intimal surface of vessels in ischemic kidneys (36).

The preliminary data collected in rat ischemic organs evidenced the capacity of this targeting approach to address activated endothelial cells of the kidney, but also on the heart and probably other organs; our attention was focused on kidneys and in particular in the prevention of tissue damages after IR.

Our failure to detect proteinuria, increased level of creatinine, and renal histologic alterations in rats undergoing IR treated with Ergidina is in line with the results of previous studies showing C involvement in the renal IRI (37–39). Evidence collected from various groups indicates that C is primarily activated through the lectin pathway in kidneys undergoing IR, as revealed by the early deposition of mannose-binding lectin (MBL) and prevention of tissue damage in MBL-deficient animals (40–42). More recently, Farrar and colleagues showed that the lectin pathway may also be triggered in ischemic kidney a few hours after reperfusion by collectin-11 found to colocalize with C3d to renal tubules (43). There has been some controversy about the contribution of the late C components to IRI through the action of C5a and/or the terminal C complex. C5b-9 was considered to play a predominant role in a mouse model of renal IRI based on the observation that C6-deficient mice were protected from tissue damage (39). Conversely, C5 inhibition failed to prevent IRI in Lewis rats raising the possibility that the involvement of the late C components in mediating renal IR may vary in different species (42). Our finding that C5 neutralization by Ergidina correlates with the prevention of renal IRI in Wistar rats does not support this hypothesis in agreement with recent data showing that antibody-mediated C5 inhibition markedly reduces tissue damage after reperfusion and prolongs graft survival in a syngeneic rat model of kidney transplantation (44).

The development of Ergidina falls within our tissue targeting approach to control C-mediated tissue damage. This strategy started with the generation of the recombinant molecule MT07 containing the C5 neutralizing antibody fused to a synovial-homing peptide that was effective at preventing joint inflammation (45). Similar to MT07, Ergidina has definite advantages over the non-targeting parent anti-C5 miniantibody that inhibits activation of circulating C5 (6). As a result of its delivery to renal endothelium, the drug guarantees local protection from the C-mediated tissue damage without affecting circulating C5 and consequently reducing the risk of infections associated with C5 depletion. This will be particularly important when treating patients with C-dependent chronic renal diseases, such as MPNG and aHUS (29), who require long-term therapy. In this case, treatment with the targeted recombinant molecule would be cost effective, since a limited amount of Ergidina (0.25 mg) corresponding to one-fourth of the dose required to partially inhibit circulating C5 (45) was sufficient to completely block the activation of C5 in the rat ischemic kidneys. The enhanced *ex vivo* binding to surgically removed kidney exposed to cold ischemia for 24 h followed by perfusion with the recombinant molecule supports its first therapeutic use to prevent posttransplant IRI, leading to delay of graft function. The contribution of C to this pathologic condition is suggested by the beneficial effect obtained perfusing the kidney with other C inhibitors including the membrane-binding C regulator APT070 (46) and, more recently, an anti-rat C5 (44) prior to transplantation. The finding that the *ex vivo* binding of Ergidina was not restricted to the kidney, but was also seen on ischemic heart, suggests that this membrane-binding anti-C5 antibody may represent a useful tool to treat surgically removed organs prior to transplantation to prevent posttransplant IRI.

In conclusion, we present a preliminary characterization of a recombinant molecule comprising a neutralizing anti-C5 antibody fused to RGD that preferentially binds *in vivo* and *ex vivo* to the ischemic vascular endothelium of surgically removed organs. This membrane-binding molecule prevents C5 activation on the cell surface and kidney injury caused by IRI.

## Ethics Statement

The *in vivo* experiments were performed in compliance with the guidelines of the European (86/609/EEC) and Italian (D.L.116/92) laws, were approved by the Italian Ministry of Health and the Administration of the University Animal House, in line with NIH Guide for the care and use of laboratory animal.

## Author Contributions

PD: performed *in vivo* experiment, composed pictures, and wrote the manuscript. DS: designed and produced vectors for protein production. SB: designed biodistribution study and analyzed data. LM: produced and characterized recombinant proteins. CG: performed biodistribution studies. GB: analyzed tissues using confocal microscopy. FC: characterized RGD interaction with the different tissues. FF: designed and analyzed functional studies. AN: designed the study and analyzed these data of the Ergidina/ischemic tissue interaction. FT and PM: designed the project, analyzed these data, and wrote the manuscript.

## Conflict of Interest Statement

AN is chairman and president of ADIENNE Pharma and Biotech, owner of Mubodina^®^ and Ergidina^®^ patent, and partially supporter of the study. All other authors declare that the research was conducted in the absence of any commercial or financial relationships that could be construed as a potential conflict of interest.
