# Supplementary figures and images for: Targeted Delivery of Neutralizing Anti-C5 Antibody to Renal Endothelium Prevents Complement-Dependent Tissue Damage
Source: Front Immunol. 2017 Sep 6;8:1093. doi: 10.3389/fimmu.2017.01093 (PMC5592221; doi:10.3389/fimmu.2017.01093)

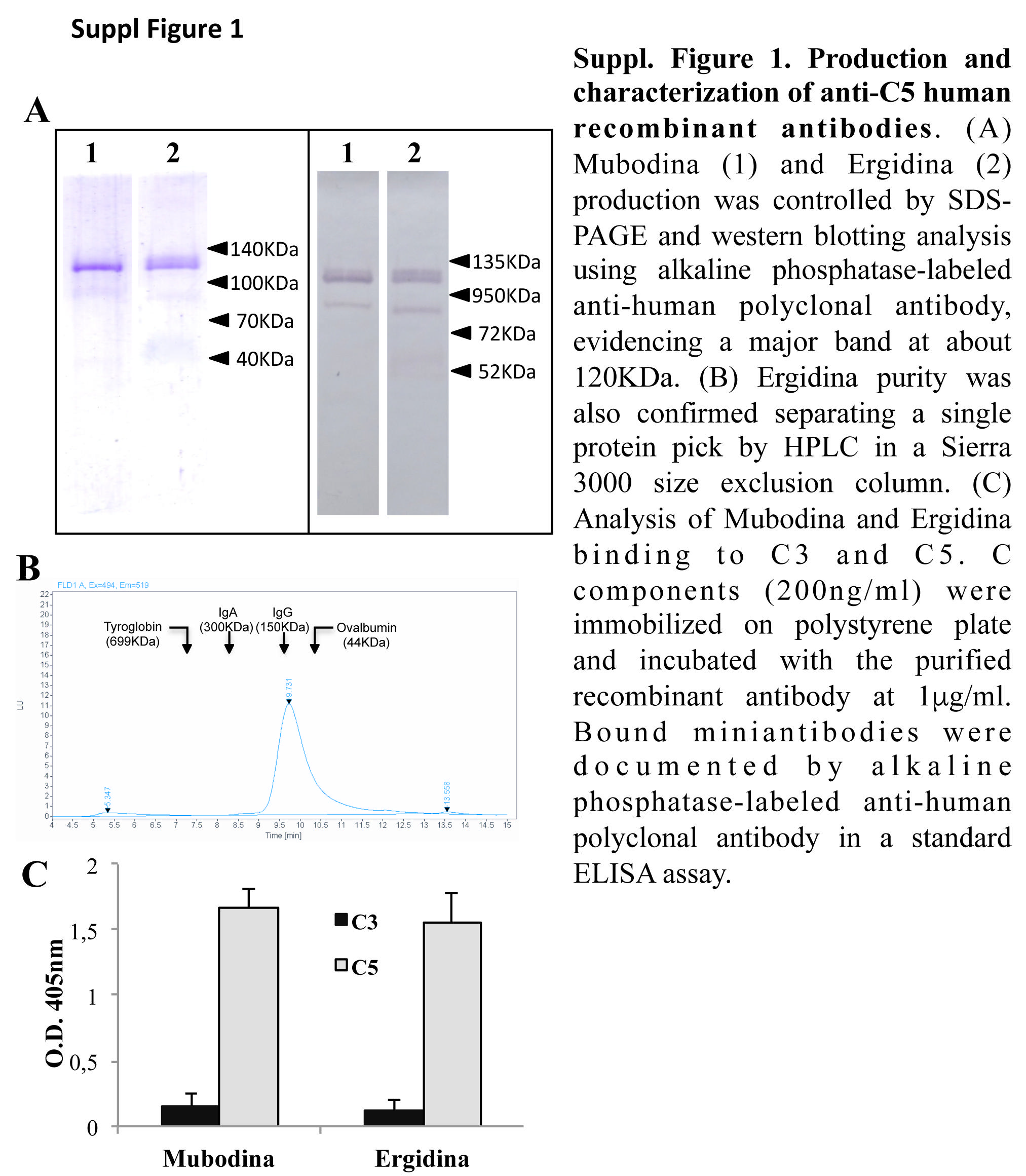

Supplement: Supplementary file 1 [file image_1.tif]

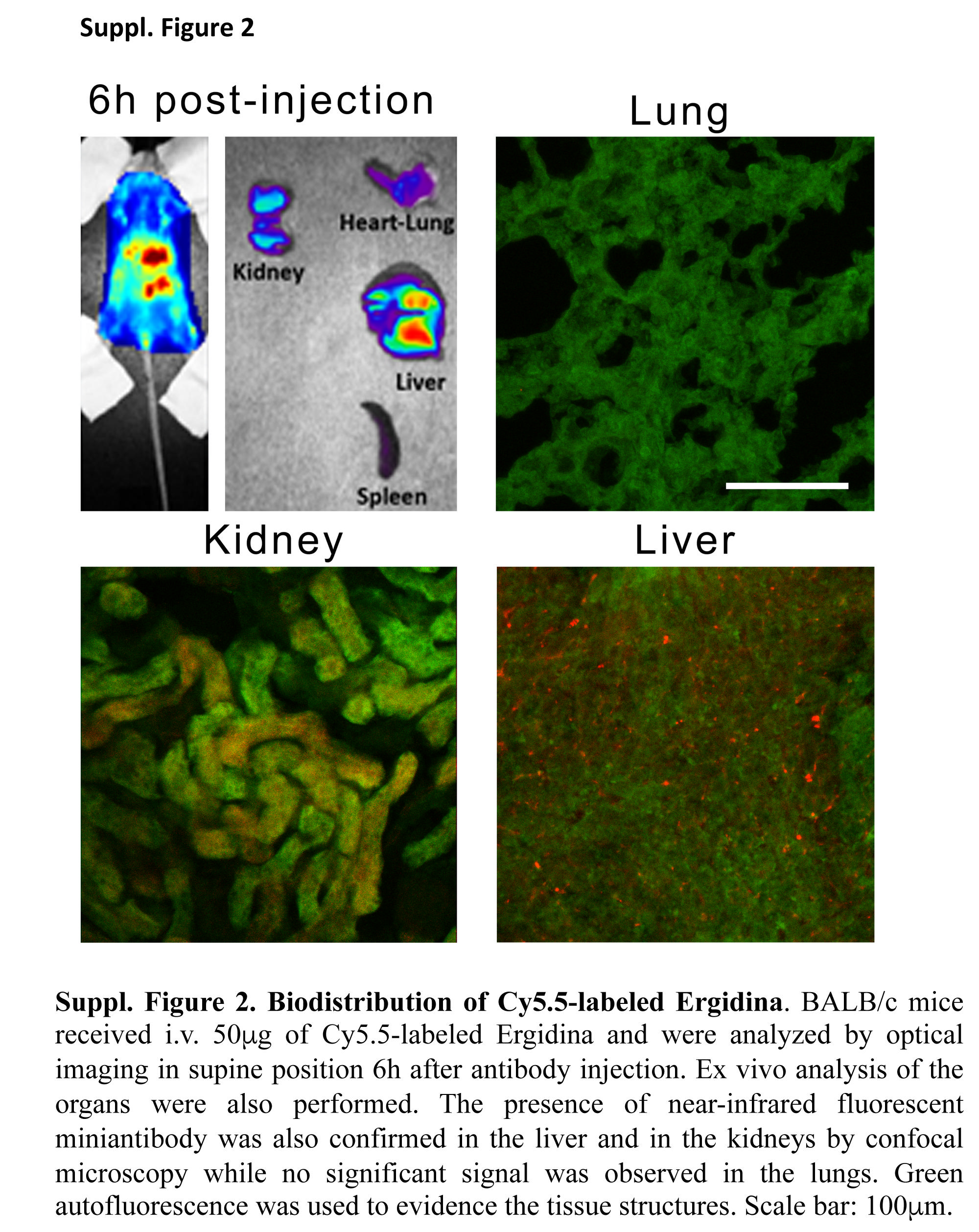

Supplement: Supplementary file 2 [file image_2.tif]

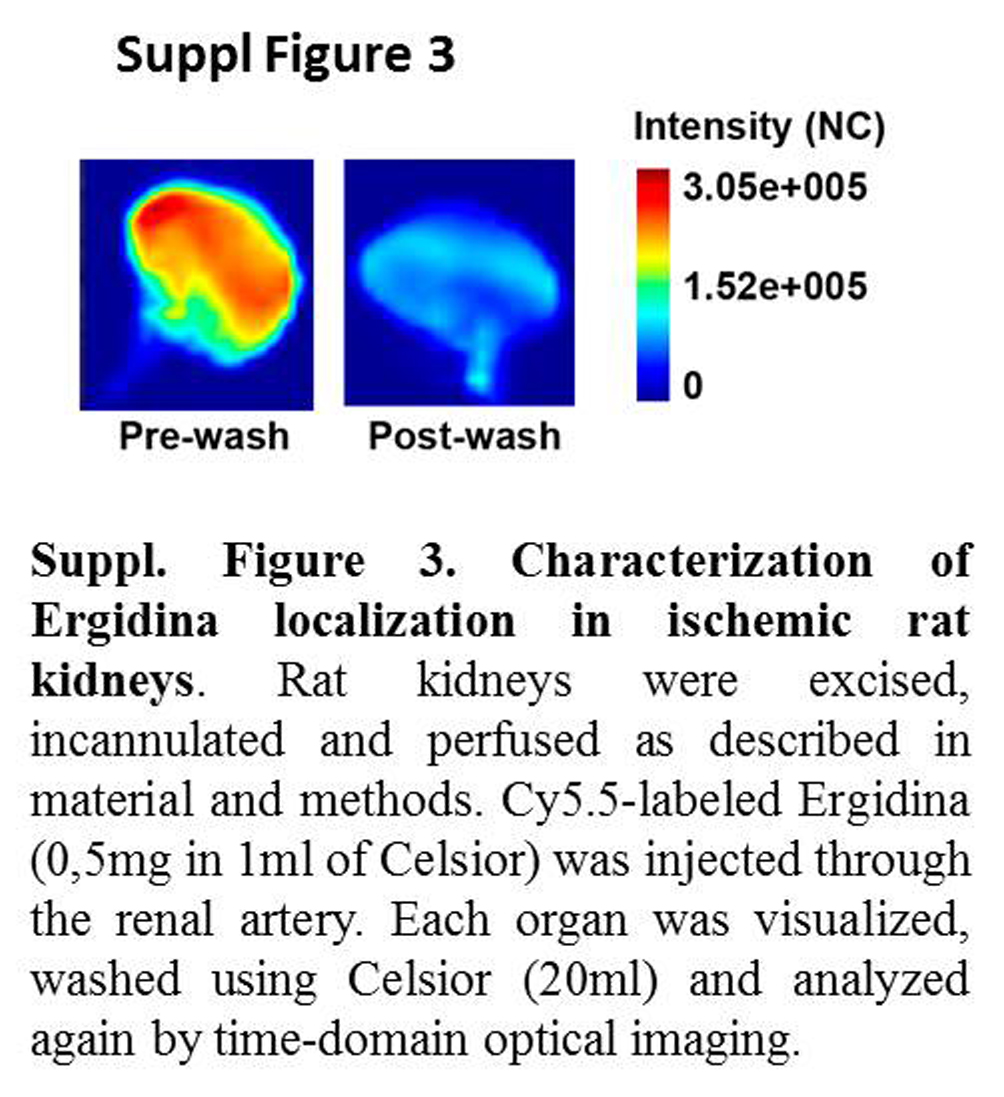

Supplement: Supplementary file 3 [file image_3.jpeg]

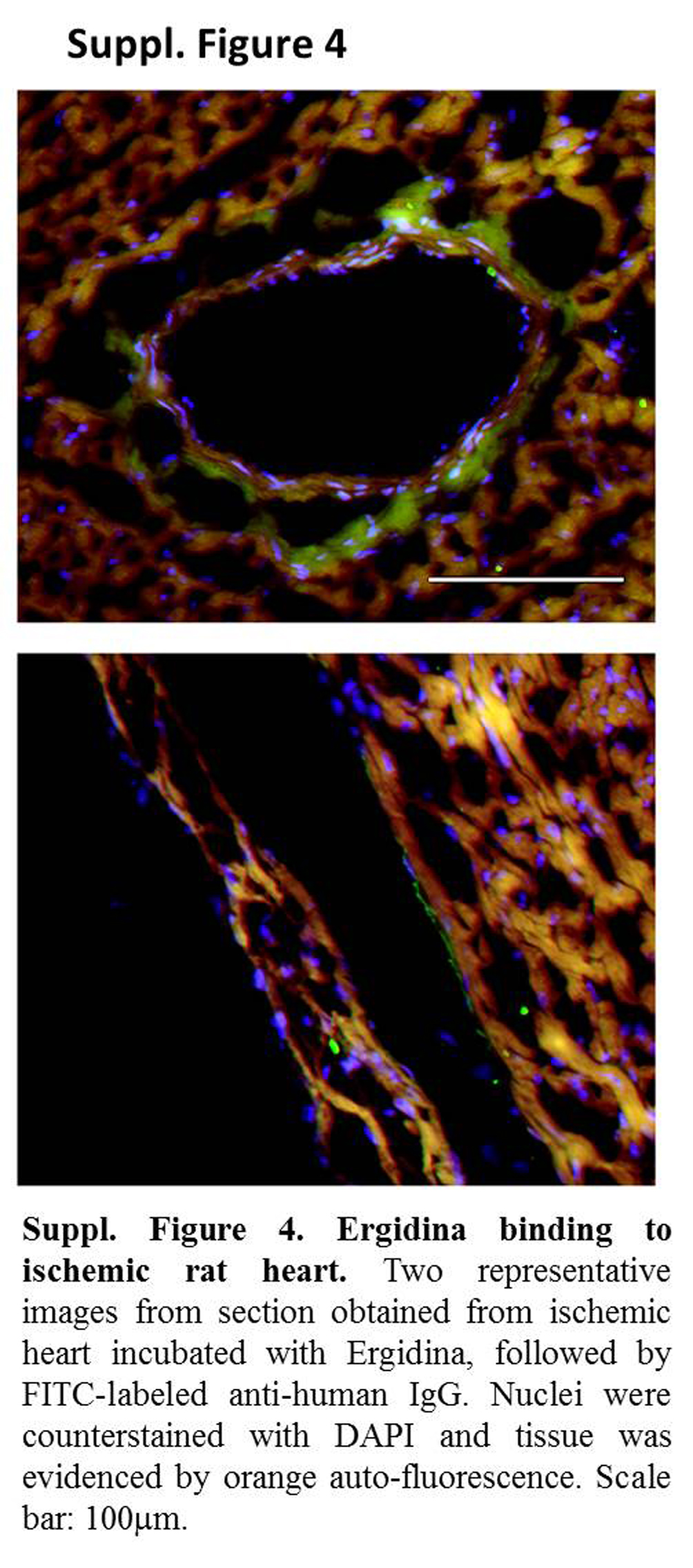

Supplement: Supplementary file 4 [file image_4.jpeg]
